# Carboligation of 5-(hydroxymethyl)furfural via whole-cell catalysis to form C12 furan derivatives and their use for hydrazone formation

**DOI:** 10.1186/s12934-023-02130-1

**Published:** 2023-06-29

**Authors:** Sara Jonsdottir Glaser, Sang-Hyun Pyo, Nicola Rehnberg, Dörte Rother, Rajni Hatti-Kaul

**Affiliations:** 1 Division of Biotechnology, Department of Chemistry, Center for Chemistry and Chemical Engineering, Lund University, 22100 Lund, Sweden; 2 Centre for Analysis and Synthesis, Department of Chemistry, Lund University, Box 124, SE-22100 Lund, Sweden; 3 Institute for Bio- and Geosciences 1: Biotechnology, Forschungszentrum Jülich GmbH, 52425 Jülich, Germany; 4 Bona Sweden AB, Box 210 74, SE-200 21 Malmö, Sweden

**Keywords:** 5-(hydroxymethyl)furfural, 5,5’-bis(hydroxymethyl)furoin (DHMF), 5,5’-bis(hydroxymethyl)furil (BHMF), Whole-cell biocatalyst, Benzaldehyde lyase, Hydrazone

## Abstract

**Background:**

Biobased 5-(hydroxymethyl)furfural (5-HMF) is an important platform that offers numerous possibilities for upgrading to a range of chemical, material and fuel products. One reaction of special interest is the carboligation of 5-HMF into C_12_ compounds, including 5,5’-bis(hydroxymethyl)furoin (DHMF) and its subsequent oxidation to 5,5’-bis(hydroxymethyl)furil (BHMF), due to their potential applications as building blocks for polymers and hydrocarbon fuels.

**Objectives:**

This study was aimed at evaluating the use of whole cells of *Escherichia coli* carrying recombinant *Pseudomonas fluorescens* benzaldehyde lyase as biocatalysts for 5-HMF carboligation, recovery of the C_12_ derivatives DHMF and BHMF, and testing the reactivity of the carbonyl groups for hydrazone formation for potential use as cross-linking agents in surface coatings. The effects of different parameters on the reaction were investigated to find the conditions for achieving high product yield and productivity.

**Results:**

The reaction with 5 g/L 5-HMF using 2 g_CDW_/L recombinant cells in 10% dimethyl carbonate, pH 8.0 at 30 °C resulted in DHMF yield of 81.7% (0.41 mol/mol) at 1 h, and BHMF yield of 96.7% (0.49 mol/mol) at 72 h reaction time. Fed-batch biotransformation generated a maximum DHMF concentration of 53.0 g/L (or 26.5 g DHMF/g cell catalyst) with productivity of 10.6 g/L^.^h, after five feeds of 20 g/L 5-HMF. Both DHMF and BHMF reacted with adipic acid dihydrazide to form hydrazone that was confirmed by Fourier-transform infrared spectroscopy and ^1^H NMR.

**Conclusion:**

The study demonstrates the potential application of recombinant *E. coli* cells for cost-effective production of commercially relevant products.

**Supplementary Information:**

The online version contains supplementary material available at 10.1186/s12934-023-02130-1.

## Introduction

The climate crisis calls for substantial decrease in global dependence on fossil feedstock to avoid the release of fixed CO_2_ into the atmosphere. As the demand for crude oil for making transportation fuel is expected to decline in the coming years, the petrochemical industry is still looking to use the raw material to make higher-margin chemicals [1]. In 2021, the petrochemical sector accounted for about 14.2% of total oil demand or 13.8 million barrels per day (mb/d) and is projected to increase to 17.5 mb/d in 2045 [2]. On the other hand, the production of chemicals from biomass has definite potential to lead to significant greenhouse gas mitigation compared to fossil-derived counterparts [3]. In addition, the use of biotransformation meets many aspects of more sustainable manufacturing besides CO_2_ reduction. For this purpose, furan platform chemicals obtained directly from C_5_ and C_6_ sugars from biomass polysaccharides are promising candidates for providing an economical route for synthesis of a vast range of bio-based chemicals, materials and fuels [4–6]. The C_6_ furan compound, 5-hydroxymethylfurfural (5-HMF), has been one of the most investigated furan compounds, both with respect to its production and its valorization to several useful building blocks for fuel, chemical, polymer and pharmaceutical industry, thanks to its reactive hydroxyl and aldehyde groups and the furan ring [7, 8]. Production of 5-HMF from fructose at quite high yields has been achieved in our laboratory using a two-phase system of water and dimethyl carbonate (DMC) in a continuous mode in a tube reactor, followed by its recovery in a pure form [9].

**Scheme 1.**
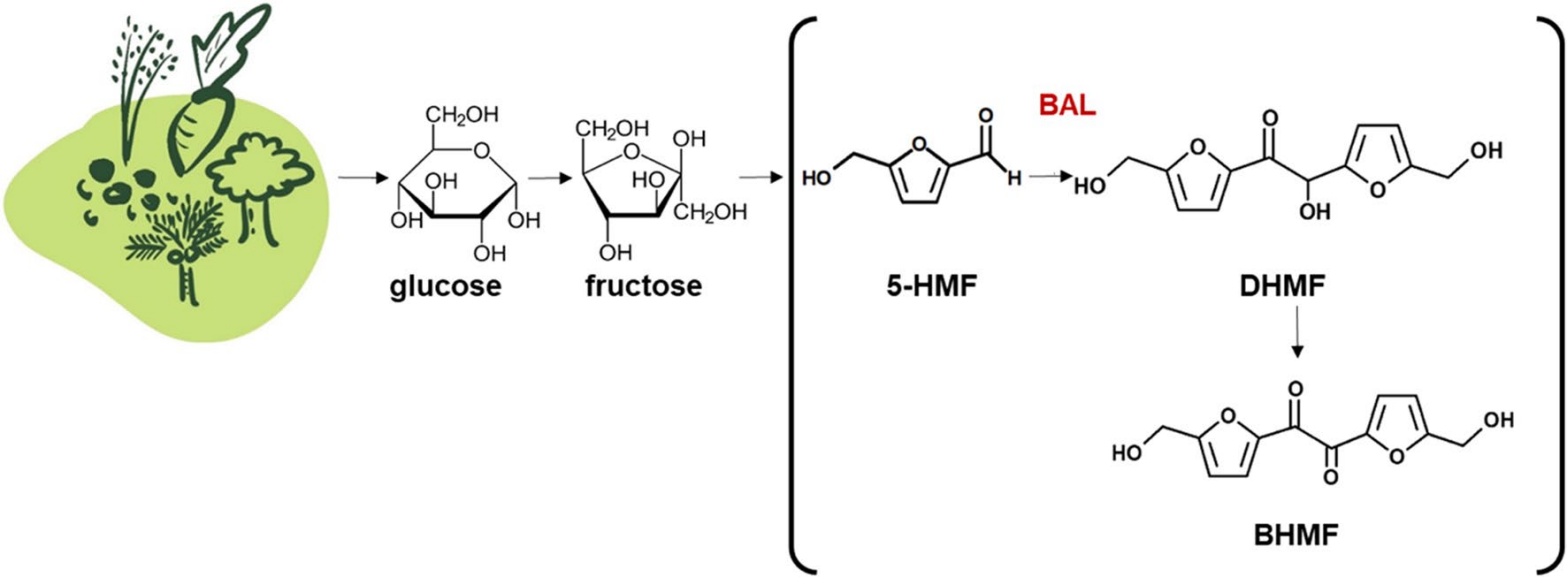
Pathway for production of C_12_ furan derivatives from sugars in the biomass via 5-HMF using recombinant *E. coli* cells expressing benzaldehyde lyase (BAL). The bracketed part is the focus of the present study

Oxidation of both aldehyde and hydroxyl groups in 5-HMF to form 2,5-furan dicarboxylic acid (FDCA), has currently attracted much attention as it is expected to serve as a biobased alternative to terephthalic acid to make polyethylene furanoate (PEF), a polymer with superior barrier and thermal properties than polyethylene terephthalate (PET) [10, 11]. Selective oxidation of the aldehyde moiety gives another interesting polymer building block, 5-hydroxymethyl-2-furancarboxylic acid (HMFCA) [12], while its reduction results in a diol, bis(hydroxymethyl)furan [13], which can be further modified e.g. to diepoxide monomers [14]. Yet another reaction that the aldehyde group has been subjected to is aldol condensation to give a variety of value added products such as fuel precursors, polymers and biologically active compounds [8, 15]. An interesting example is a carboligation reaction in which two 5-HMF molecules undergo self-condensation in an umpolung fashion to yield C_12_ acyloin (2-hydroxyketone) product, 5,5’-bis(hydroxymethyl)furoin (DHMF) [16] (See Scheme 1). DHMF has three hydroxyl groups, two furan rings and one carbonyl group, all of which can be utilised for polymerisation into polyethers, polyesters, polycarbonates and polyurethanes, production of C_12_ ketones, and/or production of oxygenated diesels [17, 18]. The 2-hydroxy ketone can be further oxidised to a diketone compound, 5,5’-bis(hydroxymethyl)furil (BHMF), in which two ketone groups connect the furan rings and has potential in producing polyurethane films [17].

The acyloin condensation reaction with 5-HMF has been done through organocatalysis using N-heterocyclic carbenes which resulted in > 95% yield of DHMF [19]. Mou et al. (2016) produced BHMF from DHMF through selective oxidation using either manganese dioxide (MnO_2_) with a high isolated yield of 95% or a “greener” metal-free organocatalyst 1,8-diazabicyclo(5–4-0)undec-7-ene (DBU) with a yield of 85% [17]. Efficient oxidation of DHMF to BHMF has also been shown using molybdenum and tungsten-based catalysts with yield up to 94% [20]. However, industrial upscaling issues remain despite good DHMF and BHMF yields via organocatalysts including stability, sensitivity and synthesis of these catalysts [21–23]. Biotransformation through enzymatic catalysis is another option for upgrading 5-HMF. Donnelly et al. have earlier shown the carboligation of 5-HMF to be catalysed by the enzyme benzaldehyde lyase from *Pseudomonas fluorescens* [24]. In another study, the same enzyme was used for the carboligation of 3-furaldehyde generating good product yields and conversion and high enantiomeric excess [25].

Benzaldehyde lyase (BAL) (E.C. 4.1.2.38) is a thiamine-diphosphate dependent enzyme which catalyses reversible conversion of (*R*)-benzoin to benzaldehyde, and is a valuable enzyme for the synthesis of chiral 2-hydroxy ketones. First reported in 1989, BAL was found to be responsible for giving *P. fluorescens* the ability to grow on benzoin as sole carbon source [26]. Due to the enzyme’s ability to catalyse C–C bond formation and high enantioselectivity, its product repertoire has since been expanded from benzoins, acetoins to hydroxybutyrophenones and aliphatic acyloins [27–29], and the insights into its three dimensional structure also opened access to stereocomplementary products thereof [30].

Despite the applicability of enzymes as drivers of selective catalysis, the high cost of enzyme purification is a major challenge for production at large scale. Production of recombinant whole cells is about ten times cheaper than isolated enzymes as it circumvents the need and costs of catalyst purification [31, 32]. Moreover, due to better stability features of whole cells, the operational lifetime of the biocatalyst is higher [31, 33].

This article investigates DHMF and BHMF production from 5-HMF in a reaction catalysed by the whole cells of recombinant *Escherichia coli* expressing *P. fluorescens* BAL. The effect of reaction parameters including co-solvent addition, varying concentrations of substrate and the biocatalyst, and other additives on the reaction performance were explored in the study. A simple purification method for purification of DHMF and BHMF was developed. Finally, reactivity of the carbonyl groups in the two products was evaluated for hydrazone formation for their potential use in crosslinking polymers and coatings.

## Results and discussion

### Screening of reaction parameters for DHMF and BHMF formation

In contrast to the earlier report demonstrating the carboligation of 5-HMF using pure BAL [24], the present work used whole cells of recombinant *E. coli* expressing benzaldehyde lyase (activity of 5071 U/g_cdw_ cells) for catalysing the reaction. SDS-PAGE analysis of protein in the cells showed a band density of 71.9% for the recombinant BAL enzyme (Additional file 1: Figure S1). Initial screening of the reaction conditions was performed in 4 mL working volume at 30 °C and the reaction was allowed to continue for 72 h. The cell catalyst was applied as resting cells resuspended in phosphate buffer (pH 8.0) at concentrations in the range of 0.2–10 g_cdw_/L. It should be noted here that the maximum DHMF and BHMF yield is 0.5 mol (100% theoretical yield) product per mol 5-HMF.

**Fig. 1:**
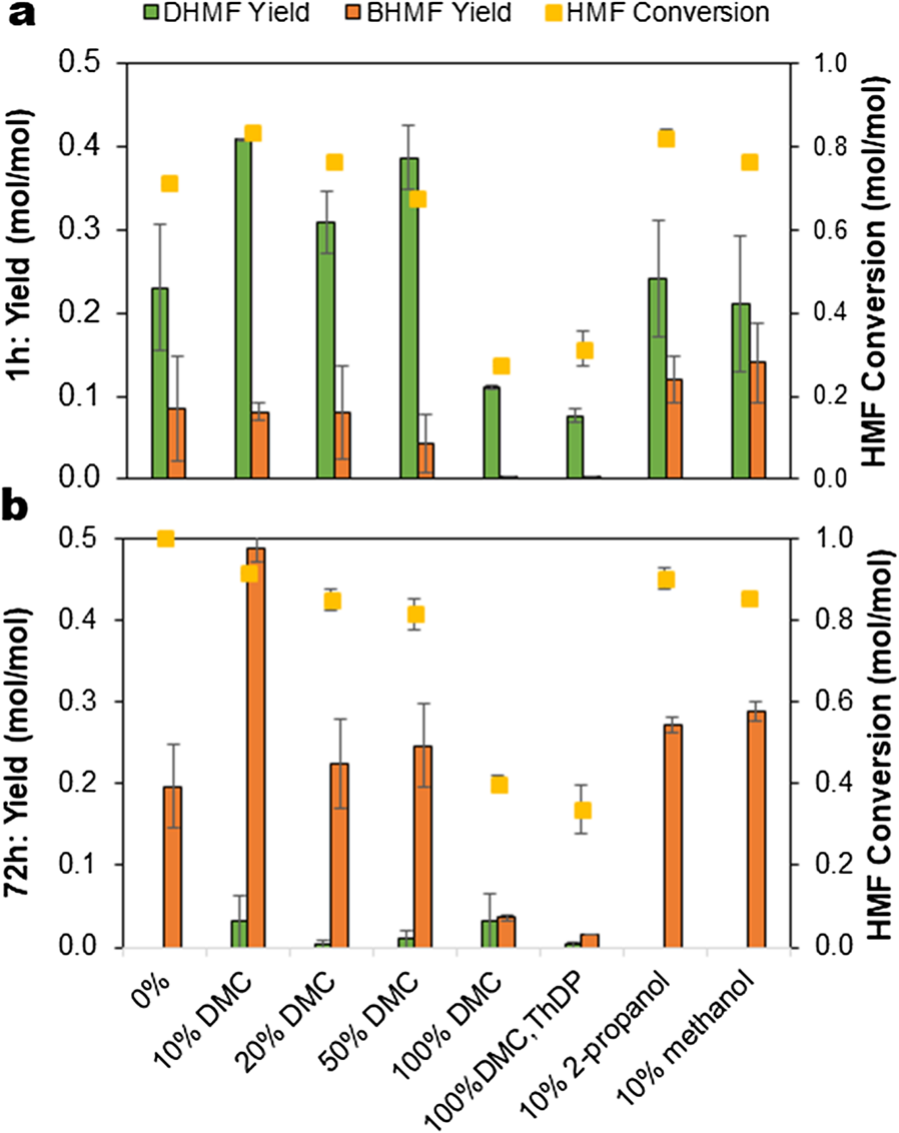
Effect of co-solvents on the yields of (**a**) DHMF, with maximum concentration at 1 h reaction time and (**b**) BHMF, with maximum concentration at 72 h reaction time during biotransformation of 5-HMF. The reaction was performed in 4 mL volume at initial substrate concentration of 5 g/L 5-HMF, with 2 g_cdw_/L recombinant *E. coli* cells expressing *P. fluorescens* benzaldehyde lyase (5071 U/g_cdw_ cells), 50 mM KH_2_PO_4_/K_2_HPO_4_ buffer pH 8.0, 2.5 mM MgSO_4_, 0.1 mM ThDP, at 30 °C and total reaction time of 72 h. Error bars indicate the standard deviation from duplicate experiments

The reaction performed in the absence of any co-solvent gave low yields of both DHMF (0.23 mol/mol) and BHMF (0.20 mol/mol), at 1 h and 72 h, respectively. The reaction using pure BAL reported earlier was performed in 20% DMSO [24], which is known to have a stabilizing effect on enzymes [34, 35] including *P. fluorescens* BAL [36, 37]. As DMSO is an undesirable solvent for large scale use due to downstream processing challenges [36] and issues related to the waste handling and safety [38], we tested other co-solvents including dimethyl carbonate (DMC), 2-propanol and methanol, which are potentially bio-based [39–41]. Previous evaluation with respect to green chemistry metrics showed that DMC and methanol are more favourable in terms of atom economy and methylating efficiency [42], while alcohols such as methanol and 2-propanol, are generally regarded as environmentally-friendly and cost-efficient [40, 43]. The highest DHMF yield of 0.41 mol/mol at 1 h and BHMF yield of 0.49 mol/mol at 72 h from 42.5 mmol (5.4 g/L) 5-HMF was obtained in a reaction with 10% (v/v) DMC (Fig. 1a and b). A blank reaction performed using *E. coli* cells without expressed BAL did not show any consumption of 5-HMF nor the formation of any product (not shown).

Adding more than 10% of DMC into the system decreased the yields of both products, which were still higher than without any solvent addition. Reactions in 10% (v/v) propanol or methanol did not have a marked influence on the DHMF yield as compared to the reaction without any solvent, but the BHMF yield was slightly increased. The higher activity of BAL in DMC containing reaction is most likely due to the relatively lower solubility of DMC in water as compared to methanol and propanol, and consequently lower impact in causing conformational changes in the enzyme. The water-miscible solvents have earlier been shown to also have a strong influence on the stereoselectivity and chemoselectivity of ThDP-dependent enzymes, the smaller the solvent molecule the higher its impact [44].

**Fig. 2:**
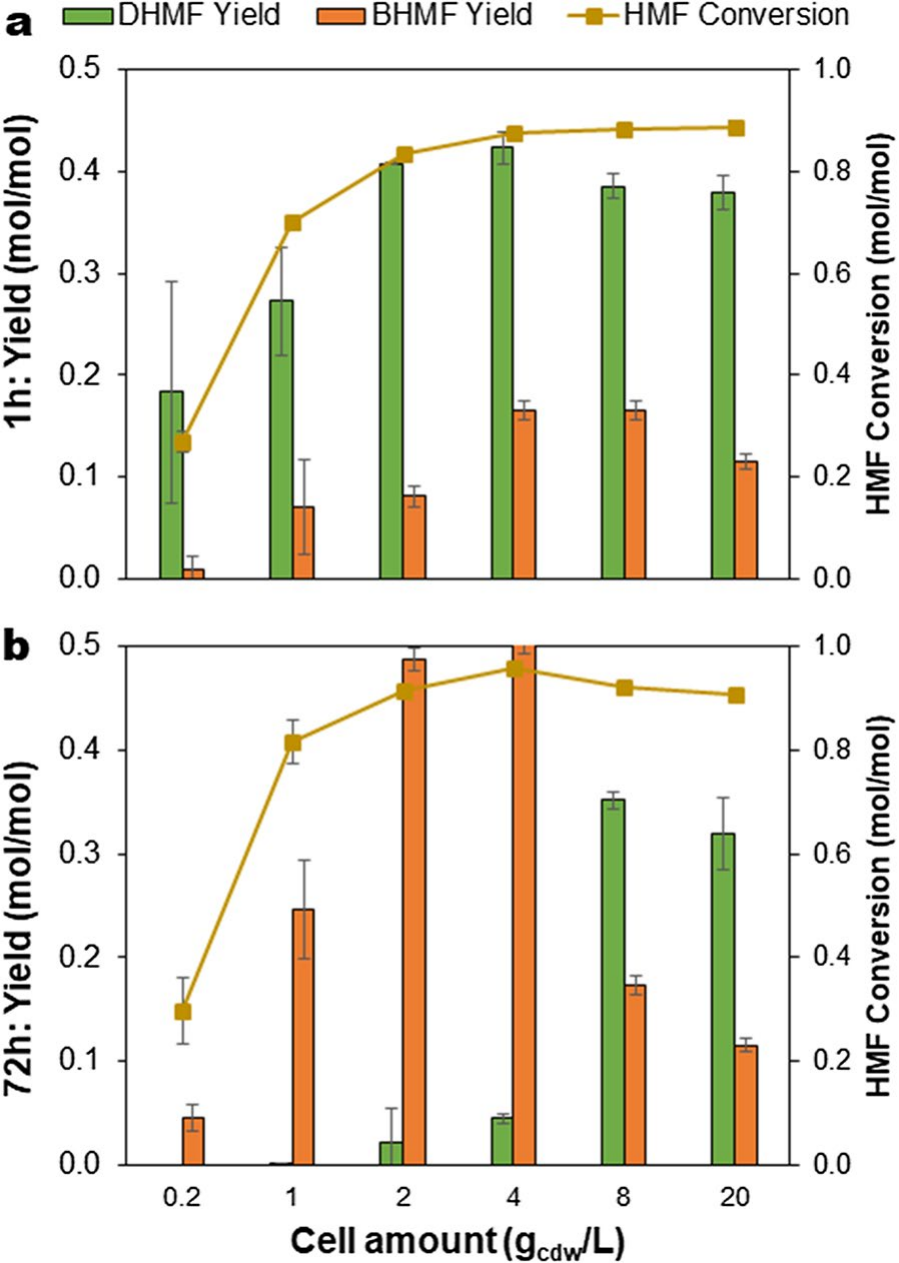
Effect of cell catalyst amount on the yields of (**a**) DHMF, with maximum concentration at 1 h reaction time and (**b**) BHMF, with maximum concentration at 72 h reaction time during biotransformation of 5-HMF. The reaction was performed in 4 mL volume using initial 5-HMF concentration of 5 g/L, 0.2–10 g_cdw_/L recombinant *E. coli* cells expressing *P. fluorescens* benzaldehyde lyase, 10% dimethylcarbonate, 50 mM KH_2_PO_4_/K_2_HPO_4_ buffer pH 8.0, 2.5 mM MgSO_4_, 0.1 mM ThDP, at 30 °C and total reaction time of 72 h. Error bars indicate the standard deviation from duplicate experiments

The reaction in 10% DMC was chosen for further experiments. Varying the concentration of cells between 0.2–10 g_cdw_/L showed maximal yields of DHMF and BHMF at 0.42 mol and 0.50 mol per mol HMF at 1 h and 72 h, respectively, at the cell concentration of 4 g_cdw_/L (Fig. 2). The yields were only marginally lower (0.41 mol and 0.49 mol DHMF and BHMF/ mol HMF, respectively) at 2 g_cdw_/L cells as also described above in Fig. 1, and this cell concentration was used for further experiments. Conversely, increasing the cell concentration to 8 g_cdw_/L and beyond also decreased the product yields. The reason for the decreased activity at higher cell concentrations is not clear but some plausible reasons could be the limited cofactor amount or changes in properties of the reaction mixture such as viscosity, cell aggregation, etc., that may affect the enzyme–substrate interaction, and hence lower activity. A tenfold decrease in productivity of lactone production with sixfold increase in the whole cell catalyst amount has been reported earlier and was suggested to be related to limitation in oxygen supply with increase in cell density [45]. Whole-cell catalysis for production of a stable ascorbic acid derivative also showed insignificant increase in product yield even with a 50% increase in the cell catalyst amount [46].

**Fig. 3:**
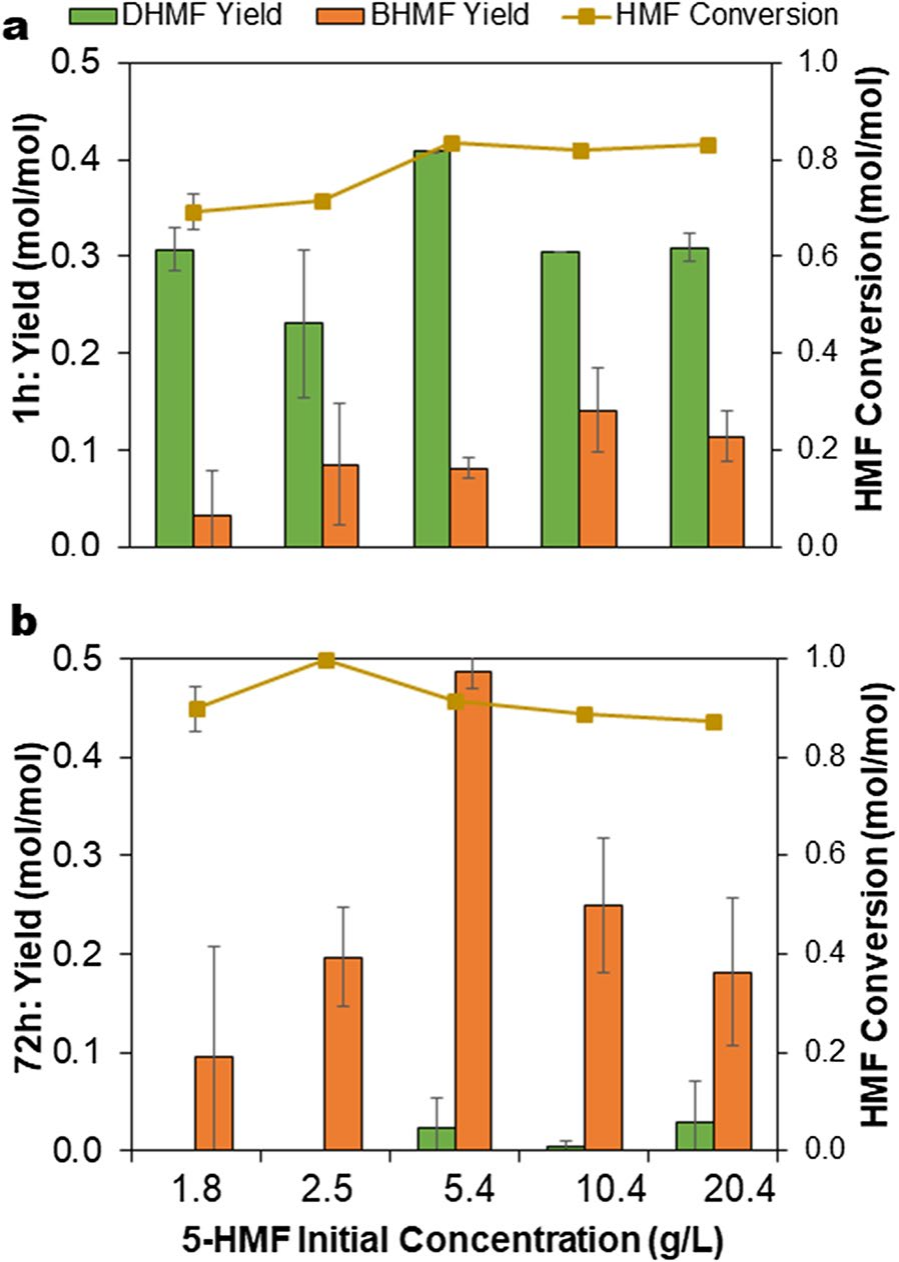
Effect of initial 5-HMF concentration on the yields of (**a**) DHMF, with maximum concentration at 1 h reaction time and (**b**) BHMF, with maximum concentration at 72 h reaction time during biotransformation of 5-HMF. The reaction was performed in 4 mL volume using initial 5-HMF concentration of 1.5–20 g/L 5-HMF, 2 g_cdw_/L recombinant *E. coli* cells expressing *P. fluorescens* benzaldehyde lyase, 10% dimethylcarbonate, 50 mM KH_2_PO_4_/K_2_HPO_4_ buffer pH 8.0, 2.5 mM MgSO_4_, 0.1 mM ThDP, temperature of 30 °C and total reaction time of 72 h. Error bars indicate the standard deviation from duplicate experiments

Reactions with varying initial 5-HMF concentration ranging from 1.5 g/L to 20 g/L in 4 mL reaction volume showed that the maximum DHMF (0.41 mol/mol) and BHMF (0.49 mol/mol) yields using 2 g_cdw_/L cell catalyst and 10% DMC were obtained at 5.4 g/L HMF (Fig. 3).

Increasing the initial substrate concentration to ~ 10 g/L and further to ~ 20 g/L decreased the DHMF yield to up to 27% and BHMF yield to 63%. However, in later experiments (See results in Fig. 5, 1st cycle), when the reaction was performed in a fed-batch mode in 10 mL working volume in a 15 mL tube, providing relatively limited aeration conditions (due to low surface-to-volume ratio), DHMF yield at 20 g/L 5-HMF increased to about 0.43 mol/mol (86% yield) after 1 h. In contrast, NHC-carbene catalysed carboligation of 5-HMF (23 g/L) performed in 5 mL tetrahydrofuran at 60 °C yielded 98% DHMF in one hour [7]. Thus, the much milder conditions provided by whole-cell catalysis can generate reasonably good DHMF yields from 5-HMF that are comparable to the organocatalytic method.

**Fig. 4:**
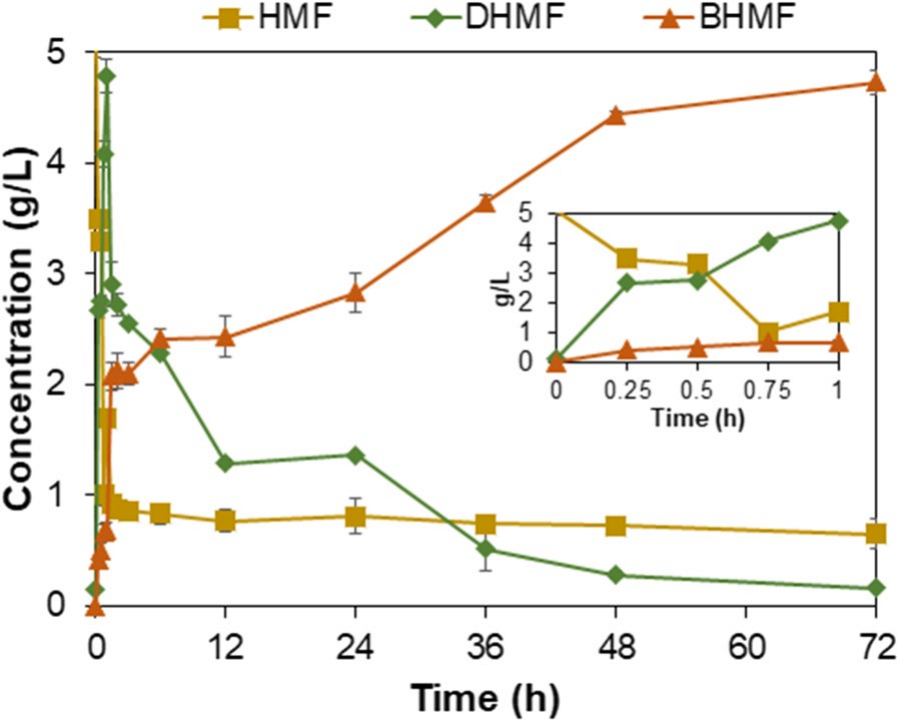
Reaction profiles of DHMF and BHMF production from 5- HMF. The reaction was performed in 4 mL volume using initial 5-HMF concentration of 5.4 g/L, 2 g_cdw_/L recombinant *E. coli* cells expressing *P. fluorescens* benzaldehyde lyase, 10% dimethylcarbonate, 50 mM KH_2_PO_4_/K_2_HPO_4_ buffer pH 8.0, 2.5 mM MgSO_4_, 0.1 mM ThDP, temperature of 30 °C, and total reaction time of 72 h. In the small plot embedded within the reaction profile, the time course of the first hour is shown in more detail. Error bars indicate the standard deviation from duplicate experiments

The reaction profile of the standard reaction with 5.4 g/L HMF in 10% DMC and 2 g_cdw_/L cells shown in Fig. 4 reveals that DHMF formation occurred quickly and peaked at 15 min (4.64 g/L) followed by a gradual transformation into BHMF reaching 4.92 g/L at 24 h and 5.19 g/L (0.49 mol /mol 5-HMF) at 72 h. BHMF formation occurs as a result of spontaneous oxidation of DHMF, which was confirmed using DHMF in a vial under identical standard conditions but excluding the *E. coli* cells. BHMF formation from DHMF with a yield of up to 0.76 mol/mol at 72 h was observed (Additional file 1: Figure S2). To test if the spontaneous oxidation of DHMF during the biocatalytic carboligation of 5-HMF could be avoided, a parallel experiment was performed in which the reaction mixture was purged with N_2_ gas in a closed reaction vessel. Under this condition, the BHMF formation was reduced to 0.24 mol BHMF per mol 5-HMF most likely due to the lower oxygen level in the system (Additional file 1: Figure S2).

On the other hand, the oxidation rate of DHMF to BHMF can be increased by increasing the aeration, as observed when the cell-catalysed 5-HMF carboligation was performed in 200 mL reaction volume in a 1 L Erlenmeyer flask, leading to a larger surface area for liquid-to-air interaction. Higher BHMF concentration was observed already at 1 h of reaction (Additional file 1: Figure S3). For efficient selective BHMF production, the crucial parameter of aeration is to be investigated carefully. Alternatively, further transformation of DHMF can be done after removal of BAL and supplementing with an efficient oxidative catalyst like reported elsewhere [17] or even using a biocatalyst, e.g. co-expression of oxidases/oxidoreductases.

### 5-HMF transformation: effects of cell recycling versus fed batch reaction

BAL is subject to inhibition by 5-HMF and probably also by the products, as observed with reduced yields at initial 5-HMF concentrations higher than 5 g/L (Fig. 3). Since the formation of DHMF occurs in a very short time, it was of interest to see if the cells retain the enzymatic activity and can be recycled for repeated transformation of 5-HMF. The biotransformation was carried out in a total volume of 10 mL with 2 g_cdw_/L cells. After running the reaction for 1 h, the cells were separated from the solution and added to a fresh reaction solution with the same 5-HMF concentration as in the previous batch. The cell recycling was repeated three times with initial 5-HMF concentrations of 5 and 10 g/L.

**Fig. 5:**
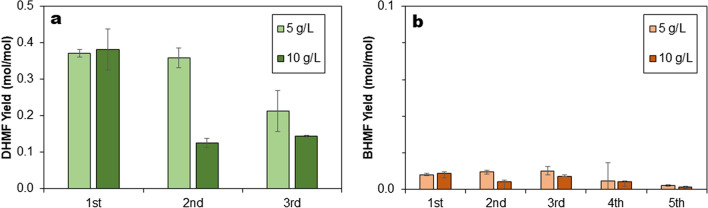
Yields (mol/mol) of **a** DHMF and **b** BHMF during cell catalyst recycling. Two separate experiments with initial 5-HMF concentrations of 5 and 10 g/L were tested. The reaction was performed for 1 h using 10% dimethylcarbonate, 2 g_cdw_/L recombinant *E. coli* cells expressing *P. fluorescens* benzaldehyde lyase, 50 mM KH_2_PO_4_/K_2_HPO_4_ buffer pH 8.0, 2.5 mM MgSO_4_, 0.1 mM ThDP, at 30 °C in 10 mL working volume. The cells were separated by centrifugation after the reaction and added to a fresh 5-HMF solution. Error bars indicate the standard deviation from duplicate experiments

As seen in Fig. 5, the yields decreased for both concentrations of 5-HMF tested after the first cycle. During the second cycle of 5 g/L 5-HMF, a marginal decrease in DHMF yield was observed from the first reaction. In comparison, the higher 5-HMF concentration (10 g/L) saw a 67% drop of DHMF yield between first and second reactions. In the third cycle, the yield decreased by 43% for 5 g/L HMF from the initial cycle, while for 10 g/L 5-HMF the yield remained about the same as in the second cycle. Further decrease in DHMF yields was observed when the cells were recycled four to five times (results not shown). The results indicate that while it may be possible to recycle the cells more than once for the production of DHMF, replenishment of the catalyst will be needed after the third cycle. In addition, the yields for BHMF remained below 0.01 mol/mol during the cell recycling experiments.

Effect of additives for reducing the enzyme inactivation and in turn improving the operational lifetime of the whole cells was investigated. The additives included 1 wt% bovine serum albumin (BSA), 2 M glycerol and 85 mM tris(hydroxymethyl)aminomethane (Tris) (Additional file 1: Figure S2). BSA and glycerol are often used as protein stabilizing agents, while Tris was used assuming that the aldehyde groups on 5-HMF may interact with the NH groups on Tris instead of the enzyme and minimize enzyme deactivation. However, none of the tested additives improved the production of DHMF (and also BHMF).

**Fig. 6:**
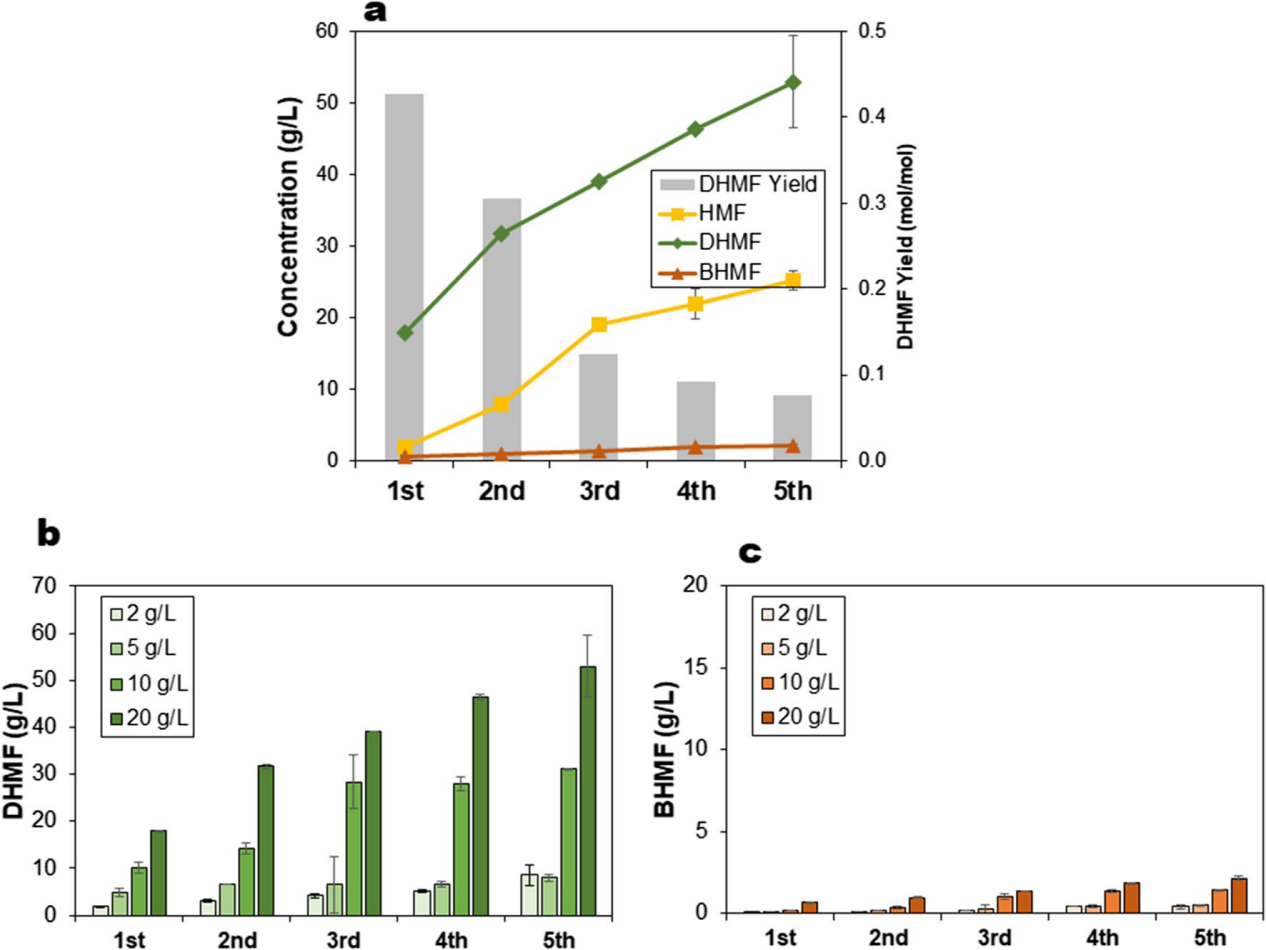
Fed-batch biotransformation of 5-HMF to DHMF in 10 mL volume using 2 g_cdw_/L recombinant *E. coli* cells expressing *P. fluorescens* benzaldehyde lyase, 10% dimethylcarbonate, 50 mM KH_2_PO_4_/K_2_HPO_4_ buffer pH 8.0, 2.5 mM MgSO_4_, 0.1 mM ThDP and temperature of 30 °C. Four separate experiments with varying initial 5-HMF concentrations of 2, 5, 10 and 20 g/L, respectively, were tested. **a** DHMF yield (mol/mol) and the corresponding concentrations of 5-HMF, DHMF and BHMF in the fed-batch experiment with 5 g/L 5-HMF feeds. **b** DHMF and **c** BHMF concentrations after each substrate feed. Error bars indicate the standard deviation from duplicate experiments

Alternatively, the biotransformation was performed in a fed-batch mode, i.e. a fresh feed of 5-HMF (2, 5, 10 and 20 g/L, respectively) was added every hour up to 5 times. Figure 6 shows increase in DHMF concentration with every feed of 5-HMF, but with decreasing yield. The final DHMF concentration obtained was 52.97 g/L from a total of 100 g/L 5-HMF (i.e. 5 feeds of 20 g/L), while BHMF concentration was 2.13 g/L. Productivity of 10.59 g/L^.^h was observed after five 20 g/L HMF feeds as compared to 17.9 g/L^.^h after the first feed (Table 1). In contrast, an earlier study by Donnelly and co-workers on the 5-HMF carboligation reaction using pure benzaldehyde lyase reports a preliminary productivity value of 7 g/L.h [24]. The highest DHMF yield of 0.43 mol/mol was obtained during the first hour, decreasing by 28% to 0.31 mol/mol during the second feed, and followed by a 70% decrease to 0.12 mol/mol, and down to 0.08 mol/mol after the fifth feed. At the end of the feed, around 24% of the total 5-HMF fed into the reactor was left unreacted. Further information on yields and productivities in both cell-recycling and fed-batch experiments are presented in Table 1**.**

**Table 1:** Yields of DHMF and BHMF per g cell catalyst and productivities during cell-recycling and fed-batch transformations with recombinant *E. coli* cells

Cycle/Feed	Yield					Yield					Productivity				
	g DHMF/g CDW					g BHMF/g CDW					g DHMF/Lh				
	1	2	3	4	5	1	2	3	4	5	1	2	3	4	5
Cell recycling															
5 g/L	2.05 ± 0.03	1.82 ± 0.05	0.92 ± 0.31	2.40 ± 2.05	0.25 ± 0.15	0.04 ± 0.00	0.05 ± 0.00	0.04 ± 0.01	0.06 ± 0.05	0.01 ± 0.00	4.10 ± 0.05	1.82 ± 0.05	0.61 ± 0.21	1.20 ± 1.02	0.10 ± 0.06
10 g/L	4.18 ± 0.54	1.39 ± 0.08	1.53 ± 0.11	1.60 ± 0.52	0.09	0.10 ± 0.01	0.05 ± 0.00	0.08 ± 0.01	0.05 ± 0.01	0.01 ± 0.00	8.37 ± 1.07	1.39 ± 0.08	1.02 ± 0.07	0.80 ± 0.26	0.03
Fed-batch															
2 g/L	0.93 ± 0.09	1.55 ± 0.10	2.09 ± 0.25	0.00	0.00	0.02 ± 0.00	0.05 ± 0.0	0.09 ± 0.01	0.21 ± 0.00	0.20 ± 0.06	1.87 ± 0.18	1.55 ± 0.10	1.39 ± 0.17	1.30 ± 0.07	1.71 ± 0.43
5 g/L	2.46 ± 0.45	3.30 ± 0.02	3.26 ± 2.97	3.26 ± 0.28	3.97 ± 0.39	0.04 ± 0.01	0.08 ± 0.01	0.14 ± 0.13	0.22 ± 0.03	0.23 ± 0.03	4.92 ± 0.91	3.30 ± 0.02	2.17 ± 1.98	1.63 ± 0.14	1.59 ± 0.16
10 g/L	5.12 ± 0.59	7.12 ± 0.60	14.20 ± 2.89	13.99 ± 0.79	15.57 ± 0.05	0.09 ± 0.01	0.17 ± 0.03	0.52 ± 0.08	0.68 ± 0.04	0.70 ± 0.03	10.24 ± 1.17	7.12 ± 0.60	9.47 ± 1.92	7.00 ± 0.40	6.23 ± 0.02
20 g/L	8.95 ± 0.10	16.03	19.50 ± 0.00	22.95	26.48 ± 3.24	0.32 ± 0.01	0.48 ± 0.01	0.69 ± 0.00	0.91 ± 0.02	1.07 ± 0.07	17.90 ± 0.20	15.85 ± 0.24	13.00 ± 0.00	11.59 ± 0.16	10.59 ± 1.30

The initial 5-HMF concentrations in the reaction during cell recycling were 5 and 10 g/L, respectively, while the concentrations were 2, 5, 10 and 20 g/L during fed-batch experiments. Both cell recycling and feeding were done 5 times at each substrate concentration. Error values indicate margin for duplicate measurements

It should be noted here that both cell-recycling and fed-batch experiments were performed without prior nitrogen sparging or tightly closed reaction vessels. However, since the 10 mL reactions were carried out in closed 15 mL Falcon tubes, except for sample withdrawal and feedings of 5-HMF, there was very limited aeration throughout the reaction. In a separate experiment where a specifically nitrogen sparged and closed vessel protocol was implemented, the product yields were similar to the reaction protocol using the 15 mL Eppendorf tubes, i.e. BHMF formation is reduced (Additional file 1: Figure S2).

However, when the working volume was increased to 200 mL and the reaction done in a 1 L Erlenmeyer flask, while maintaining identical reaction parameters (2 g_cdw_/L cell catalyst and 5 g/L HMF) and feeding the same substrate amount every hour, the BHMF production increased within one hour (Additional file 1: Figure S3). Interestingly, the proportion of BHMF being produced in 200 mL working volume at 1 h of reaction was much higher compared to the 10 mL fed-batch experiments, and even more so than the initial 4 mL experiments, suggesting the conversion of DHMF to BHMF to be promoted by the larger surface area for oxygen transfer.

### Recovery of DHMF and BHMF, and hydrazone formation

Both DHMF and BHMF were extracted from the reaction solution using ethyl acetate; most DHMF could be extracted after 1 h of the biotransformation and then recovered as a pure white substance after evaporation of the extract and dissolving the solid residue in ethyl acetate-heptane mixture. Recovery of pure BHMF was achieved using a similar procedure and separating the supernatant from the white residue of DHMF followed by evaporation. ^1^H NMR analyses of the purified DHMF and BHMF showed 96.7% and 98.1% purity, respectively (Additional file 1: Figure S4).

While the potential use of DHMF and BHMF as building blocks for different polymers and also for forming deep-eutectic solvents has already been proposed [24], the utilisation of the functional keto groups in these compounds to generate crosslinks would be valuable for the enhanced performance of the products e.g. polyurethane coatings. The reactivity of the keto groups in the 2-hydroxyketone and diketone compounds was evaluated by reaction with adipic acid dihydrazide at room temperature to generate hydrazone crosslinks (Additional file 1: Scheme S1). With a molar ratio of 2 mol DHMF/BHMF to 1 mol adipic acid dihydrazide in 50:50 methanol:water, the product formed was completely soluble in the solvent after mixing vigorously for one minute.

**Fig. 7:**
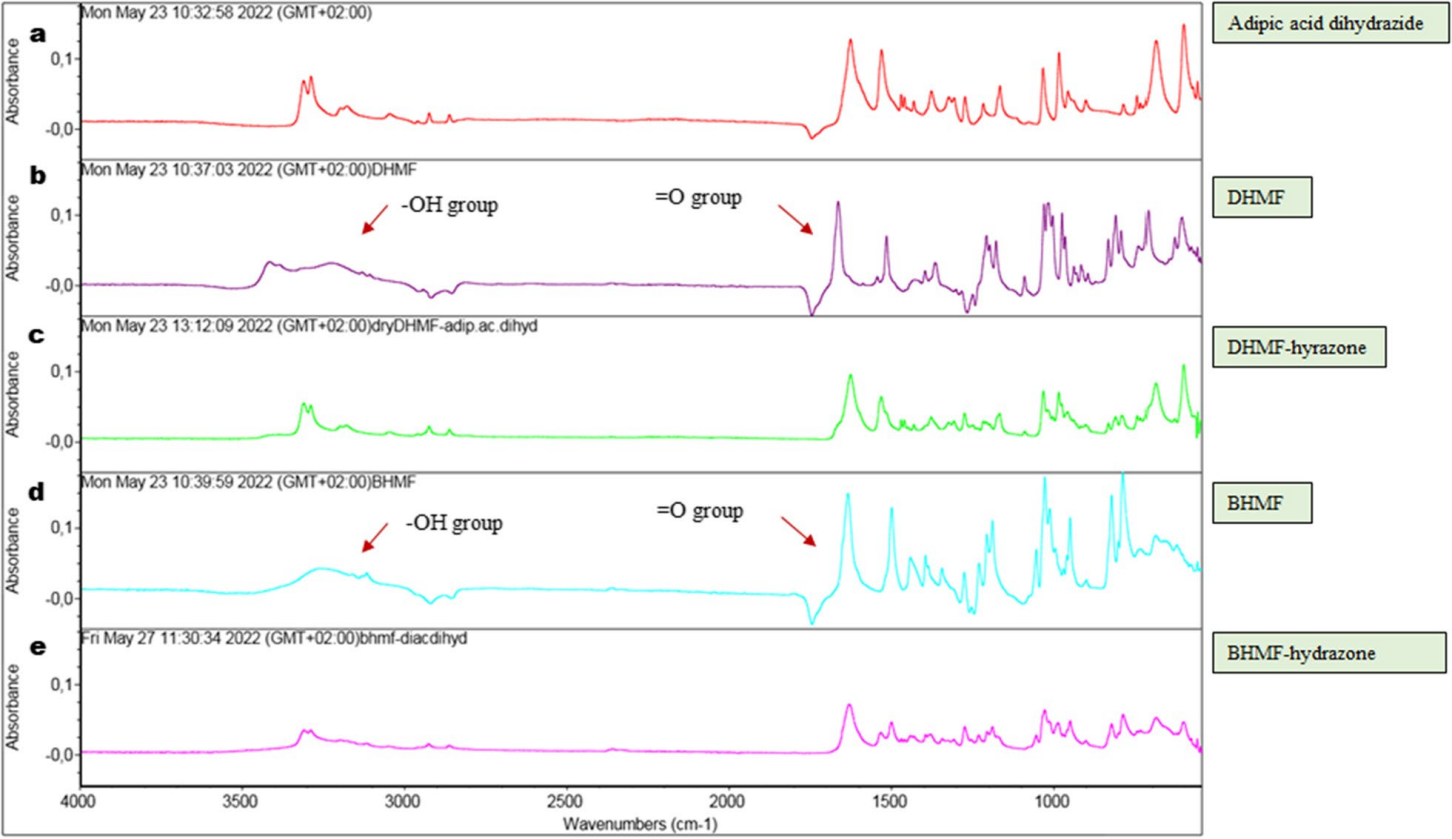
FTIR absorbance spectra of hydrazone formation by reaction of DHMF and BHMF, respectively, with adipic acid dihydrazide for 2 h

The FTIR spectra of the product sample showed a change of signal at about 1700 cm^−1^ signifying C = N bond formation (Fig. 7c, e**),** in contrast to the corresponding spectrum of DHMF and BHMF with the relatively narrow peaks at about1600 cm^−1^** (**Fig. 7b, d**)**. The C = N bond formation characterising a hydrazone, results from a substitution reaction between the keto group in DHMF with the amide substituent of the hydrazide. Changes in the OH^−^ group peaks (3200–3400 cm^−1^) were also apparent before and after reaction with adipic acid hydrazide to form hydrazones. The ^1^H NMR spectra of the products formed further validate the formation of hydrazones as seen by a decrease in the − NH_**2**_ peak (4.15 ppm) (Additional file 1: **Figure S5**). The BHMF-based hydrazone sample showed nearly complete disappearance of the − NH_2_ peak (4.15 ppm), whilst the N–H peak (8.93 ppm) remained visible indicating complete reaction of the dihydrazide with BHMF (Additional file 1: Figure S5c). On the other hand, DHMF-based hydrazone showed a lower − NH_**2**_ peak relative to the N–H peak due to an incomplete hydrazone formation (Additional file 1: Figure S5b). Since this reaction is pH-dependent [47, 48], and this preliminary test was done in an unbuffered aqueous methanol solution, further optimisation of the reaction is recommended.

## Conclusion and outlook

This study has shown the whole microbial cells bearing benzaldehyde lyase to be an effective biocatalyst for carboligation of 5-HMF in a very short time under ambient conditions. Up to 52.7 g DHMF /L (26.5 g DHMF per g cells) was obtained during fed-batch conditions involving 20 g/L 5-HMF feeds. Performing the reaction with limited access to oxygen increased DHMF formation at higher 5-HMF initial concentrations, while also decreasing the formation of the furil BHMF. Cell recycling for repeated reactions as well as fed-batch reaction showed decreasing product yields with successive cycles/feeds due to inactivation of the enzyme activity. The potential of the keto groups in DHMF/BHMF for hydrazone formation was also demonstrated, suggesting their possible role as crosslinking agents in polymer coatings and gels.

Further work should focus on increasing the kinetics of BHMF production and upscaling the process whilst maintaining high substrate conversion and good product yield and recovery. Additionally, evaluation of the product as crosslinker in coatings is planned.

## Materials and methods

### Materials

5-HMF was procured from Nanjing Confidence Chemical Co. Dimethyl carbonate, methanol, 2-propanol, dipotassium hydrogen phosphate, potassium dihydrogen phosphate, thiamine diphosphate, magnesium sulfate and heptane were purchased from Sigma Aldrich Merck. Ethyl acetate was from VWR Chemicals. All solvents used were of analytical grade.

### Production of the whole cell catalyst

Plasmid pET28A containing the gene encoding the enzyme BAL with 6-His tag (between the restriction sites by NcoI and XhoI) was obtained from Forschungszentrum Jülich, Germany [27], and was transformed into *Escherichia coli* BL21 using the Hanahan protocol [49]. *E. coli* BL21 pET28A BAL was then cultivated in 250 mL Autoinduction (AI) medium (containing 10 g/L tryptone, 0.15 g/L MgSO_4_, 6.8 g/L KH_2_PO_4_, 0.5 g/L glucose. 5 g/L yeast extract, 3.3 g/L (NH_4_)_2_SO_4_, 7.1 g/L Na_2_HPO_4_, 2 g/L α-lactose) in 500 mL Erlenmeyer flasks at 20 °C, 250 rpm for 24 h (Infors HT Ecotron, Switzerland) to reach final OD_600_ of 5.5. The culture broth was centrifuged at 3392×g, 4 °C for 30 min (Sigma 3-16PK, Germany). The supernatant was discarded and cells were frozen at -20 °C until further use. To prepare the whole cell catalyst for DHMF production, the frozen cells, 70.2 mg cell dry weight (CDW), were resuspended in 1 mL of 50 mM KH_2_PO_4_/K_2_HPO_4_ buffer pH 8.0, supplemented with 2.5 mM MgSO_4_ and 0.1 mM thiamine diphosphate.

### Biotransformation of 5-HMF to DHMF and BHMF

In a typical reaction, 10 g/L 5-HMF was added to 50 mM KH_2_PO_4_/K_2_HPO_4_ buffer, 2.5 mM MgSO_4_, 0.1 mM thiamine diphosphate, pH 8.0 with 10% co-solvent in a total reaction volume of 4 mL. The cell suspension prepared as described above was added to the mixture at a concentration of 0.2–10 g_cdw_/L. The reaction vials were incubated at 30 °C in a thermomixer (Hettich Benelux, Switzerland) set at 600 rpm for up to 72 h. The co-solvents tested were dimethylcarbonate, methanol and 2-propanol, respectively. Fifty microliter samples were withdrawn at regular time intervals for analysis of products and residual substrate.

The fed-batch experiments were done using similar concentrations of reaction components except for a larger working volume, 10 mL in 15 mL falcon tubes and 200 mL in a 1 L Erlenmeyer flask, respectively. At one hour intervals, 10 g/L 5-HMF was added to the reaction vessel. The tubes were incubated at 30 °C with shaking at 250 rpm (Infors HT Ecotron, Switzerland).

The cell-recycling experiments were also performed in 10 mL working volume, while maintaining the concentration of reaction components as above. At one hour intervals, the cells were separated by centrifugation at 3392 × g, 4 °C, 10 min (Sigma 3-16PK, Germany). The cells were then reused to catalyse a fresh reaction. This was repeated for five cycles. The biotransformation reactions, fed-batch and cell-recycling experiments were done in duplicates.

### Purification of DHMF and BHMF

DHMF and/or BHMF were extracted six times from the reaction liquid using ethyl acetate at 1:1 ratio. The organic phase was pooled together and concentrated using a rotavapor (Heidolph, Germany) until a whitish powder was formed. Ethyl acetate was added to the dried material to facilitate the removal of the product from the evaporator flask. Heptane was then mixed with ethyl acetate at a ratio of 35:65 mL until precipitation of pure DHMF occurred. The supernatant containing BHMF was separated from the precipitate. Pure BHMF was obtained by evaporating the supernatant.

### Analyses

#### Cell density and cell dry weight determination

Cell density was monitored by measuring absorbance of a cell suspension at 600 nm. Cell dry weight (CDW) was measured gravimetrically by pipetting 1 mL of cell solution into a pre-incubated (24 h, 105 °C) and pre-weighed Eppendorf tube. The cells were separated from the supernatant through centrifugation for 3 min at 13 000 rpm (Eppendorf AG, Hamburg, Germany). The cells were washed twice with 1 mL of 0.9% NaCl solution and separated again by centrifugation. Subsequently, 1 mL distilled water was added for resuspension and then incubated for 24 h at 105 °C. Prior to weighing, the tubes with the dried cells were left at a dessicator for at least 1 h. The weight of the dried cells (CDW) was then correlated to the OD of samples taken at the same time (CDW/OD = 0.233; standard deviation = 0.034).

#### Protein analysis

Total protein determination was performed by bicinchoninic acid (BCA) assay using the manufacturer´s protocol (Pierce™ BCA Protein assay kit, Themo Scientific™). Bovine serum albumin (BSA) was used as protein standard.

SDS-PAGE was performed on a commercially prepared stain-free 12% polyacrylamide gel (Bio-Rad) and electrophoresed at 250 mV for 40 min using Mini-Protean Tetra Electrophoresis Cell (Bio-Rad). GelDoc Go Imaging system (Bio-Rad) was used to scan the gel.

#### BAL activity determination

BAL activity was determined using a coupled photometric assay in which the benzaldehyde formed by the enzyme catalysed cleavage of benzoin, is reduced by NADH dependent horse liver alcohol dehydrogenase (HL-ADH) to benzyl alcohol. The reaction was followed by monitoring the decrease in concentration of NADH (standard equation: Abs = 2.0711 (mM NADH) + 0.311) as a function of time at 340 nm. It was ensured that the BAL concentration is the rate limiting step.

1 $$Benzoin~~ \to 2~benzaldehyde~\frac{{HL - ADH}}{{NADH \to NAD^{ + } }} \to ~~2~benzylalcohol~~$$

The assay mixture consisted of 850 µL reaction buffer containing 1.72 mM *rac*-benzoin, 85 vol% buffer (50 mM KH_2_PO_4_/K_2_HPO_4_ buffer, 2.5 mM MgSO_4_, 0.1 mM thiamine diphosphate, pH 8.0), 15 vol% PEG400) with 50 µL of 7 mM NADH, 50 µL of 5.5 mM HL-ADH, and equilibrating at 30 °C for 5 min. This was followed by addition of 50 µL BAL solution and measuring the absorbance at 340 nm for 90 s. The enzyme acitivity can be calculated as follows:

2 $$Activity \left( {{\raise0.7ex\hbox{$U$} \!\mathord{\left/ {\vphantom {U {mL}}}\right.\kern-0pt} \!\lower0.7ex\hbox{${mL}$}}} \right) = {\raise0.7ex\hbox{${dA}$} \!\mathord{\left/ {\vphantom {{dA} {min}}}\right.\kern-0pt} \!\lower0.7ex\hbox{${min}$}}*\frac{V}{{v*d*\left( {2\varepsilon_{p} } \right)}} = \frac{dA}{{min}}*1.6077$$

where *U* denotes enzyme units (µmol/min); dA is change of absorption at 340 nm per min, *V* is the total volume (1 mL); *v* is the volume of cell suspension sample (50 µL); ε_p_ is extinction coefficient of NADH (6.31 L mmol^−1^ cm^−1^), taking into account that one benzoin molecule is cleaved into two benzaldehyde molecules [27]. One unit (U) of benzaldehyde lyase activity is defined as the amount of the enzyme needed to catalyse the cleavage of 1 µmol benzoin per minute under standard conditions of 30 °C and 1 atm.

#### Product analysis via HPLC

5-HMF, DHMF and BHMF were analysed using High Performance Liquid Chromatography (HPLC, Jasco, Japan) equipped with a reversed-phase chromatography column (C_18_ Kromasil, Sweden) connected to a C_18_ guard column (Kromasil, Sweden). The chromatography was performed at column temperature of 30 °C, using 20–80% (v/v) methanol gradient at 0.6 mL/min, and monitored by UV detection at 280 nm. Prior to analysis, the reaction samples were diluted at least 1:5 with 20% methanol. The diluted samples were separated from solid particles by passing through 0.2 µm filters. A representative chromatogram for the quantitative analysis of 5-HMF, DHMF and BHMF is presented in Additional file 1: Figure S6.

#### Product analysis via FTIR and ^1^H NMR

Fourier Transform Infrared Spectroscopy (FTIR) of the DHMF, BHMF and hydrazone samples were carried out in a Nicolet 6700 spectrometer (Thermo Fischer Scientific, Waltham, MA, USA). The structure and purity of DHMF and BHMF as well as the hydrazone products were verified by ^1^H NMR (DMSO-d_6_) using Varian Inova 400 MHz spectrometer. Tetramethylsilane was used as an internal reference standard for determination of chemical shifts (ppm) in the ^1^H NMR spectra.

#### Hydrazone formation

Two moles of DHMF or BHMF were mixed vigorously with 1 mol adipic acid dihydrazide in 50:50 methanol:water for about one minute until the reagents were fully dissolved. The mixture was left to air-dry for about two hours. The product was analysed using FTIR and ^1^H NMR.

## Supplementary Information

**Additional file 1**: **Figure S1. **SDS-PAGE gel of the E. coli cells expressing benzaldehyde lyase. Lane 1=cell suspension, lane 2=insoluble fraction, lane 7= Precision Plus protein ladder, lane 12=soluble fraction. The total protein content of the cell suspension was 3.6 mg/mL. BAL molecular weight= ~49 kDa. **Figure S2**. Effect of various additives on the yields ofDHMF yield as green bars, with maximum concentration at 1h reaction timeBHMF yield as orange bars, with maximum concentration at 72 h reaction time. The reaction was performed using initial 5-HMF concentration of 2 g/L, 2 gcdw/L recombinant E. coli cells expressing P. fluorescens benzaldehyde lyase, 10 % dimethylcarbonate, 50 mM KH2PO4/K2HPO4 buffer pH 8.0, 2.5 mM MgSO4, 0.1 mM ThDP, temperature 30 °C and total reaction time of 72 h. The yellow points indicate 5-HMF conversion. BSA=bovine serum albumin, TRIS=tisaminomethane, N2-purged=nitrogen purged for 5 minutes prior to start of reaction. The last columnshows BHMF yield mol per mol DHMF. **Figure S3**. Fed-batch biotransformation in 200 mL working volume in 1 L flask, with 5 g/L 5-HMF initial concentration, 2 gcdw/L cells, 10% dimethylcarbonate, 30°C, 50 mM KH_2_PO_4_/K_2_HPO_4_ buffer pH 8.0, 2.5 mM MgSO_4_, 0.1 mM ThDP. 5-HMFwas fed every 1 hour. **Figure S4. **^1^H NMR of purifiedDHMF andBHMF. **Figure S5. **^1^H NMR spectra of hydrazone formation from DHMF and BHMF with adipic acid dihydrazide.Adipic acid dihydrazide,DHMF-based hydrazone, andBHMF-based hydrazone. **Figure S6. **Representative chromatogram for quantitative analysis of 5-HMF, DHMF and BHMF by HPLC using reversed phase columnand 20–80% methanol/water as mobile phase at 30°C, and UV detection at 280 nm. The retention times were 7.2 min, 9.4 min and 16.2 min for 5-HMF, DHMF anf BHMF, respectively. **Scheme S1**. Hydrazone formation via reaction with adipic dihyrazide.DHMF-based hydrazone, andBHMF-based hydrazone.

## Data Availability

The data sets supporting the results presented in this publication are included within the article and the supplementary file. The datasets used and/or analyzed during the current study are available from the corresponding author on request.
