# Supplementary material for: Carboligation of 5-(hydroxymethyl)furfural via whole-cell catalysis to form C12 furan derivatives and their use for hydrazone formation
Source: Microb Cell Fact. 2023 Jun 29;22:120. doi: 10.1186/s12934-023-02130-1 (PMC10311727; doi:10.1186/s12934-023-02130-1)
Supplement: Supplementary file 1 — Additional file 1: Figure S1. SDS-PAGE gel of the E. coli cells expressing benzaldehyde lyase. Lane 1=cell suspension, lane 2=insoluble fraction, lane 7= Precision Plus protein ladder, lane 12=soluble fraction. The total protein content of the cell suspension was 3.6 mg/mL. BAL molecular weight= ~49 kDa. Figure S2. Effect of various additives on the yields ofDHMF yield as green bars, with maximum concentration at 1h reaction timeBHMF yield as orange bars, with maximum concentration at 72 h reaction time. The reaction was performed using initial 5-HMF concentration of 2 g/L, 2 gcdw/L recombinant E. coli cells expressing P. fluorescens benzaldehyde lyase, 10 % dimethylcarbonate, 50 mM KH2PO4/K2HPO4 buffer pH 8.0, 2.5 mM MgSO4, 0.1 mM ThDP, temperature 30 °C and total reaction time of 72 h. The yellow points indicate 5-HMF conversion. BSA=bovine serum albumin, TRIS=tisaminomethane, N2-purged=nitrogen purged for 5 minutes prior to start of reaction. The last columnshows BHMF yield mol per mol DHMF. Figure S3. Fed-batch biotransformation in 200 mL working volume in 1 L flask, with 5 g/L 5-HMF initial concentration, 2 gcdw/L cells, 10% dimethylcarbonate, 30°C, 50 mM KH2PO4/K2HPO4 buffer pH 8.0, 2.5 mM MgSO4, 0.1 mM ThDP. 5-HMFwas fed every 1 hour. Figure S4. 1H NMR of purifiedDHMF andBHMF. Figure S5. 1H NMR spectra of hydrazone formation from DHMF and BHMF with adipic acid dihydrazide.Adipic acid dihydrazide,DHMF-based hydrazone, andBHMF-based hydrazone. Figure S6. Representative chromatogram for quantitative analysis of 5-HMF, DHMF and BHMF by HPLC using reversed phase columnand 20–80% methanol/water as mobile phase at 30°C, and UV detection at 280 nm. The retention times were 7.2 min, 9.4 min and 16.2 min for 5-HMF, DHMF anf BHMF, respectively. Scheme S1. Hydrazone formation via reaction with adipic dihyrazide.DHMF-based hydrazone, andBHMF-based hydrazone. [file 12934_2023_2130_MOESM1_ESM.docx]

Supplementary Information

**Carboligation of 5-(Hydroxymethyl)furfural via Whole-Cell catalysis to Form C12 Furan Derivatives and Their Use for Hydrazone Formation**

Sara Jonsdottir Glaser^a^, Sang-Hyun Pyo^a^, Nicola Rehnberg^b^, Dörte Rother^c^, Rajni Hatti-Kaul^a,*^

^a^Division of Biotechnology, Department of Chemistry, Center for Chemistry and Chemical Engineering, Lund University, SE-22100 Lund, Sweden

^b^Bona AB and Polymer Chemistry, Department of Chemistry, Center for Chemistry and Chemical Engineering, Lund University, SE-22100 Lund, Sweden

^c^ IBG-1: Biotechnology, Forschungszentrum Julich GmbH, 52425 Jülich, Germany

^*^*Corresponding author*

Tel: +46-46-222 4840

E-mail: rajni.hatti-kaul@biotek.lu.se


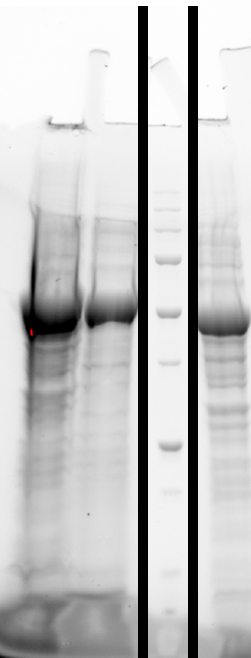


**BAL**

**BAL**

**250-**

**150-**

**100-**

**75-**

**50-**

**37-**

**25-**

**20-**

**15-**

**10-**

**-250**

**-150**

**-100**

**-75**

**-50**

**-37**

**-25**

**-20**

**-15**

**-10**

**1 2 7 12**

**Figure S1.** SDS-PAGE gel of the *E. coli* cells expressing benzaldehyde lyase (BAL). Lane 1=cell suspension, lane 2=insoluble fraction, lane 7= Precision Plus protein ladder (Bio-Rad, 10 – 250 kDa), lane 12=soluble fraction. The total protein content of the cell suspension was 3.6 mg/mL. BAL molecular weight= ~49 kDa.

DHMF

N_2_-purged 3.5 g_cdw_/L

N_2_-purged 2 g_cdw_/L

**b**

**a**

**Figure S2.** Effect of various additives on the yields of (a) DHMF yield as green bars, with maximum concentration at 1h reaction time (b) BHMF yield as orange bars, with maximum concentration at 72 h reaction time. The reaction was performed using initial 5-HMF concentration of 2 g/L, 2 g_cdw_/L recombinant *E. coli* cells expressing *P. fluorescens* benzaldehyde lyase, 10 % dimethylcarbonate, 50 mM KH_2_PO_4_/K_2_HPO_4_ buffer pH 8.0, 2.5 mM MgSO_4_, 0.1 mM ThDP, temperature 30 °C and total reaction time of 72 h. The yellow points indicate 5-HMF conversion (mol/mol). BSA=bovine serum albumin, TRIS=tis(hydroxymethyl)aminomethane, N_2_-purged=nitrogen purged for 5 minutes prior to start of reaction. The last column (yellow) shows BHMF yield mol per mol DHMF.

**Figure S3.** Fed-batch biotransformation in 200 mL working volume in 1 L flask, with 5 g/L 5-HMF initial concentration, 2 g_cdw_/L cells, 10% dimethylcarbonate, 30°C, 50 mM KH_2_PO_4_/K_2_HPO_4_ buffer pH 8.0, 2.5 mM MgSO_4_, 0.1 mM ThDP. 5-HMF (5 g/L) was fed every 1 hour.


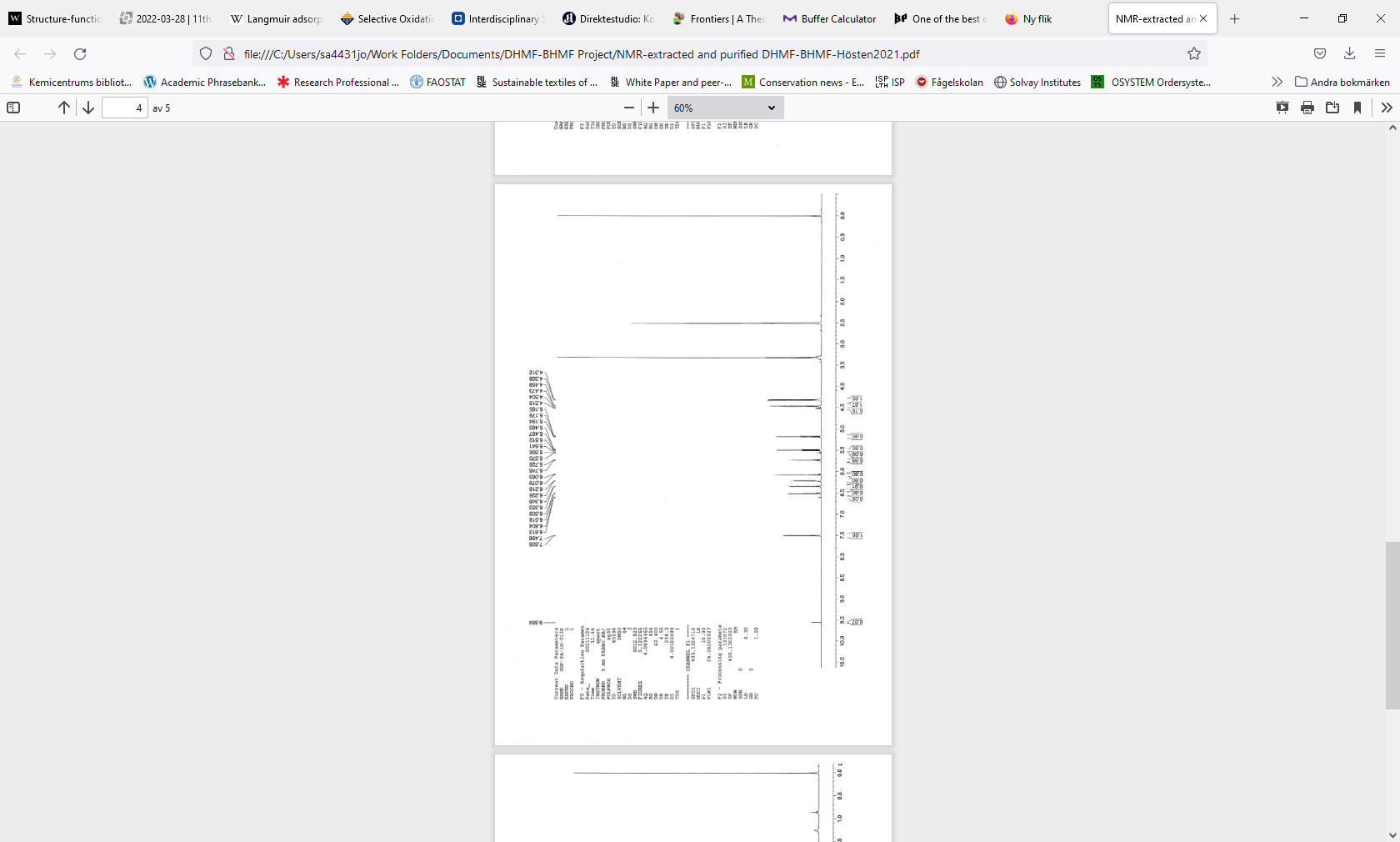


**a**


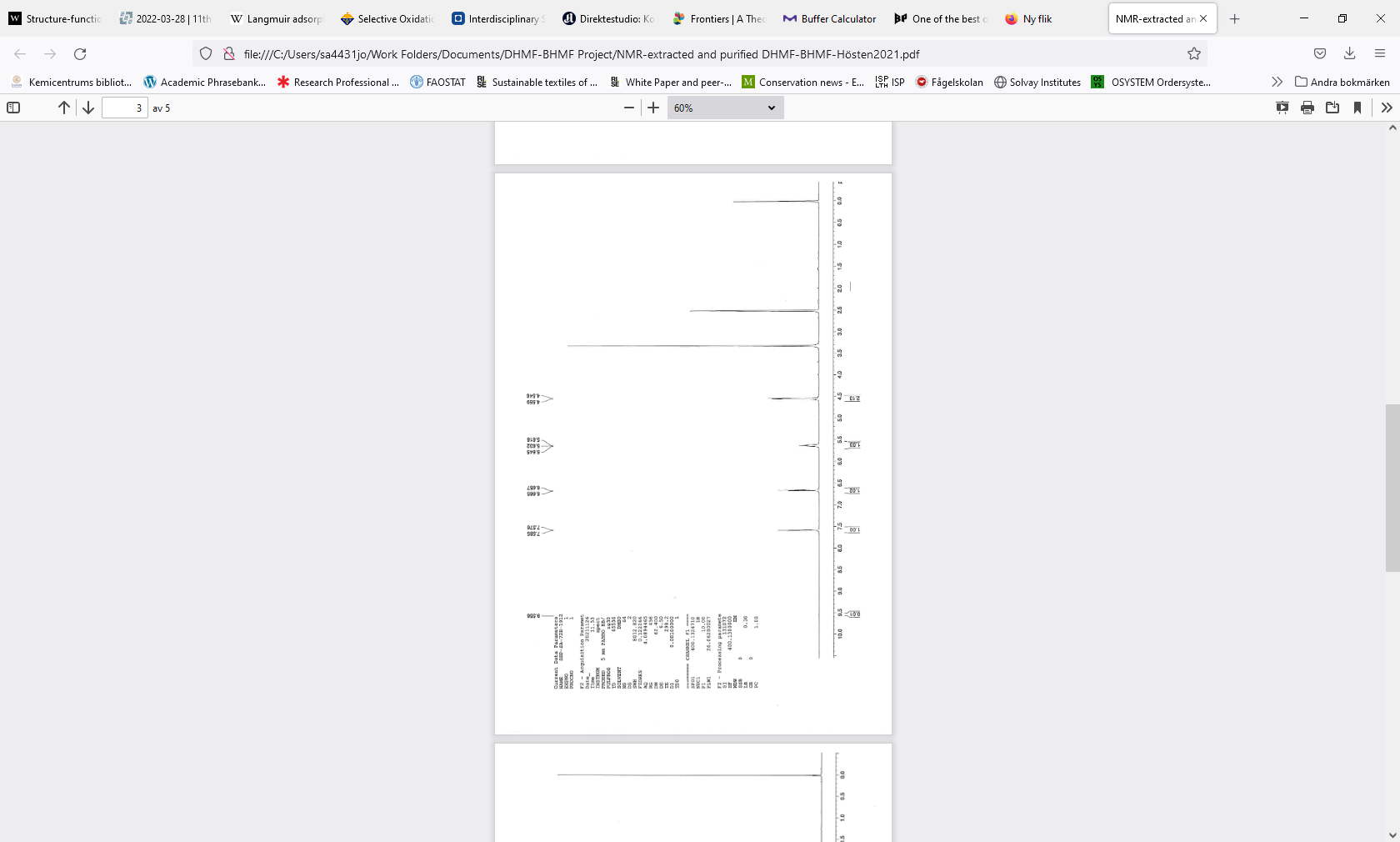


**b**

**Figure S4.** ^1^H NMR of purified (a) DHMF and (b) BHMF


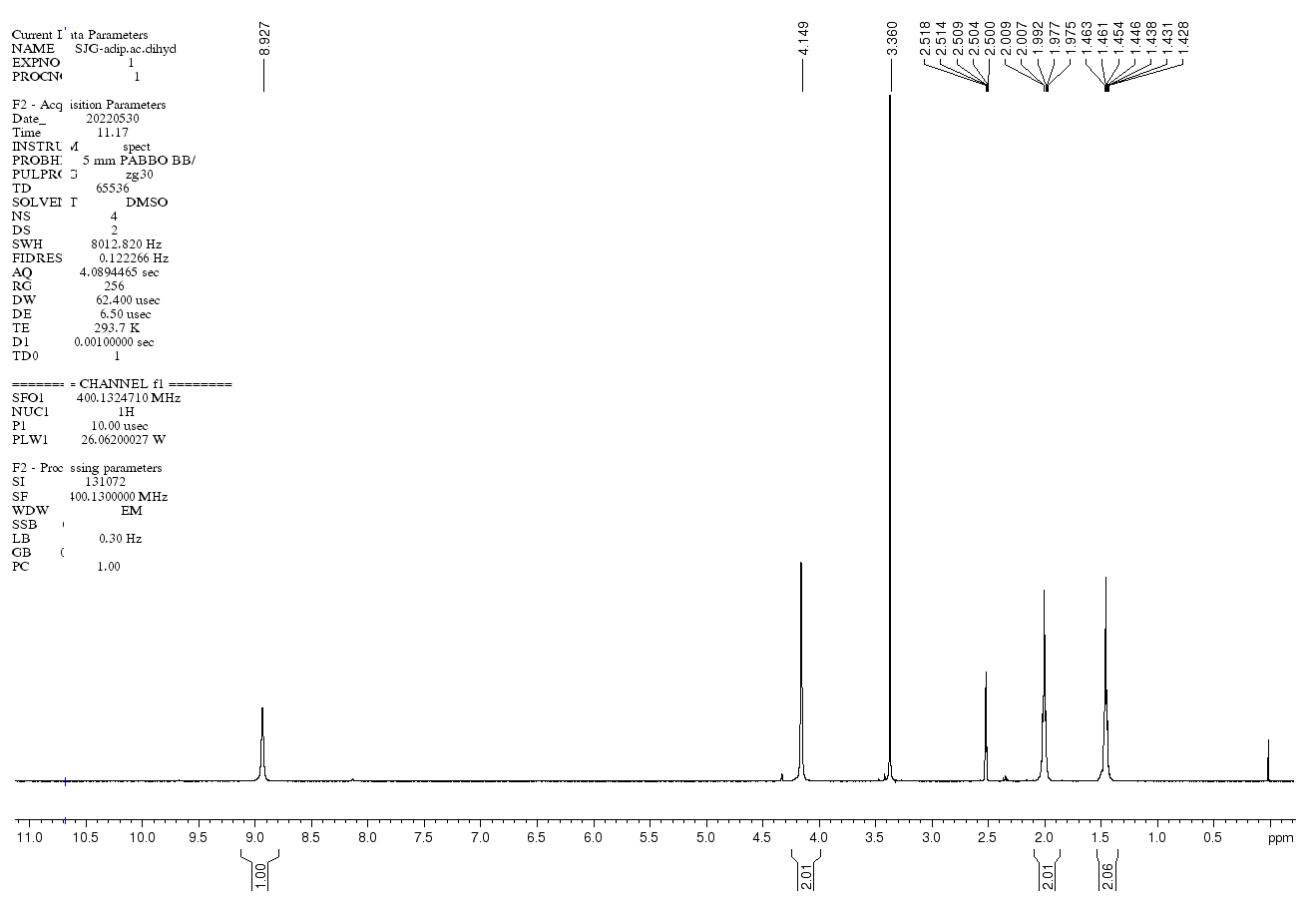


**a**


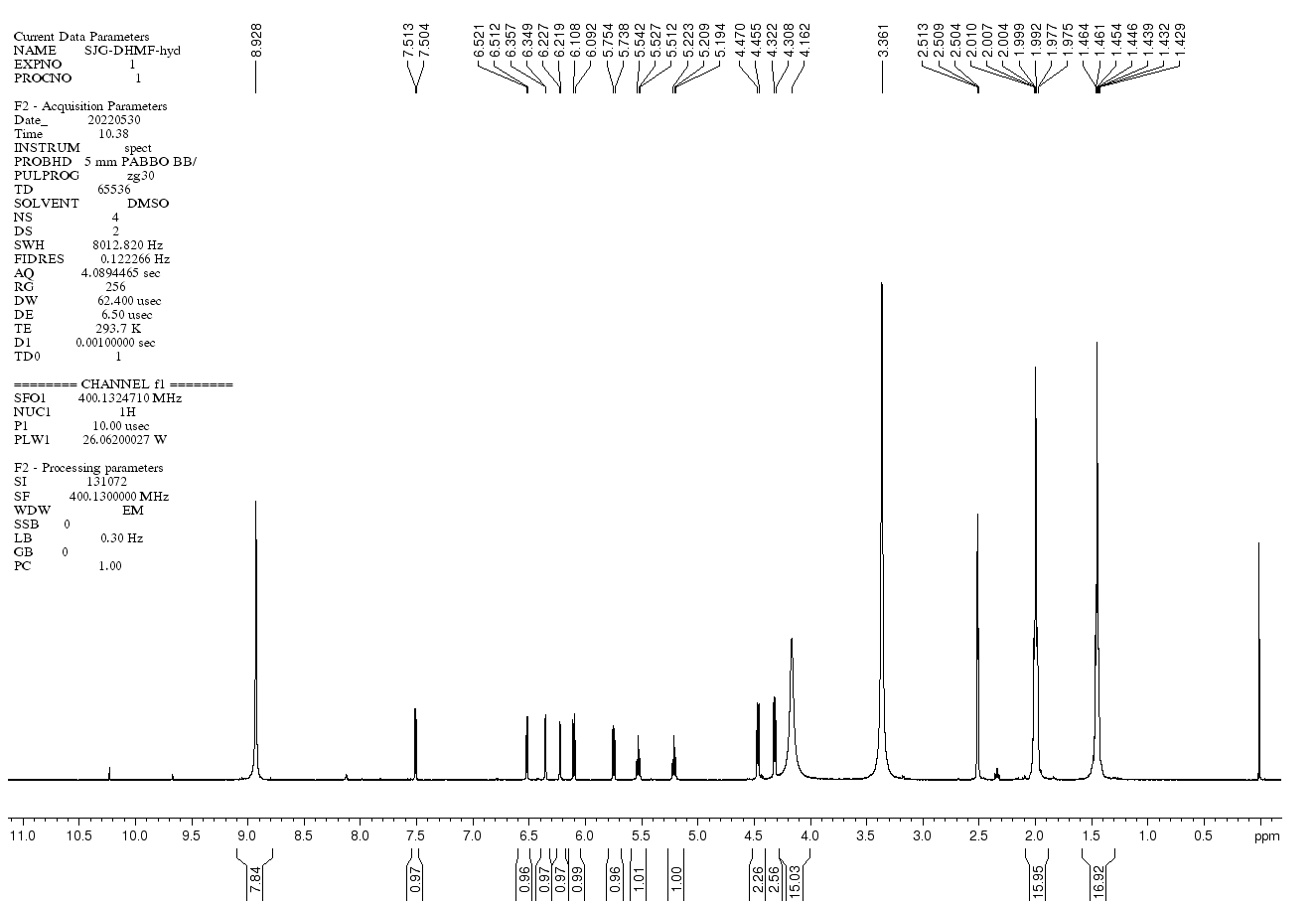


**b**


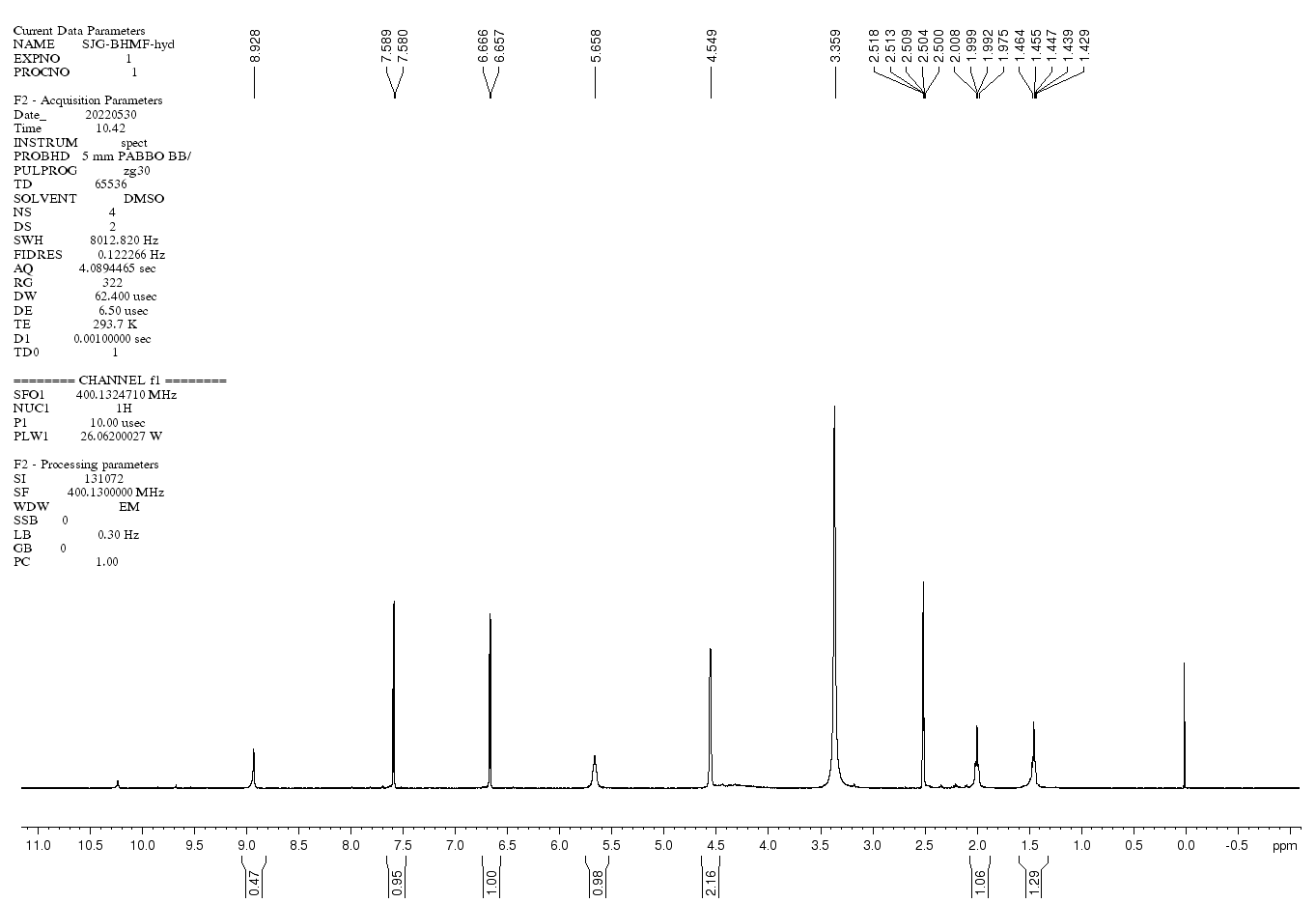


**c**

**Figure S5.** ^1^H NMR spectra of hydrazone formation from DHMF and BHMF with adipic acid dihydrazide. (a) Adipic acid dihydrazide, (b) DHMF-based hydrazone, and (c) BHMF-based hydrazone.


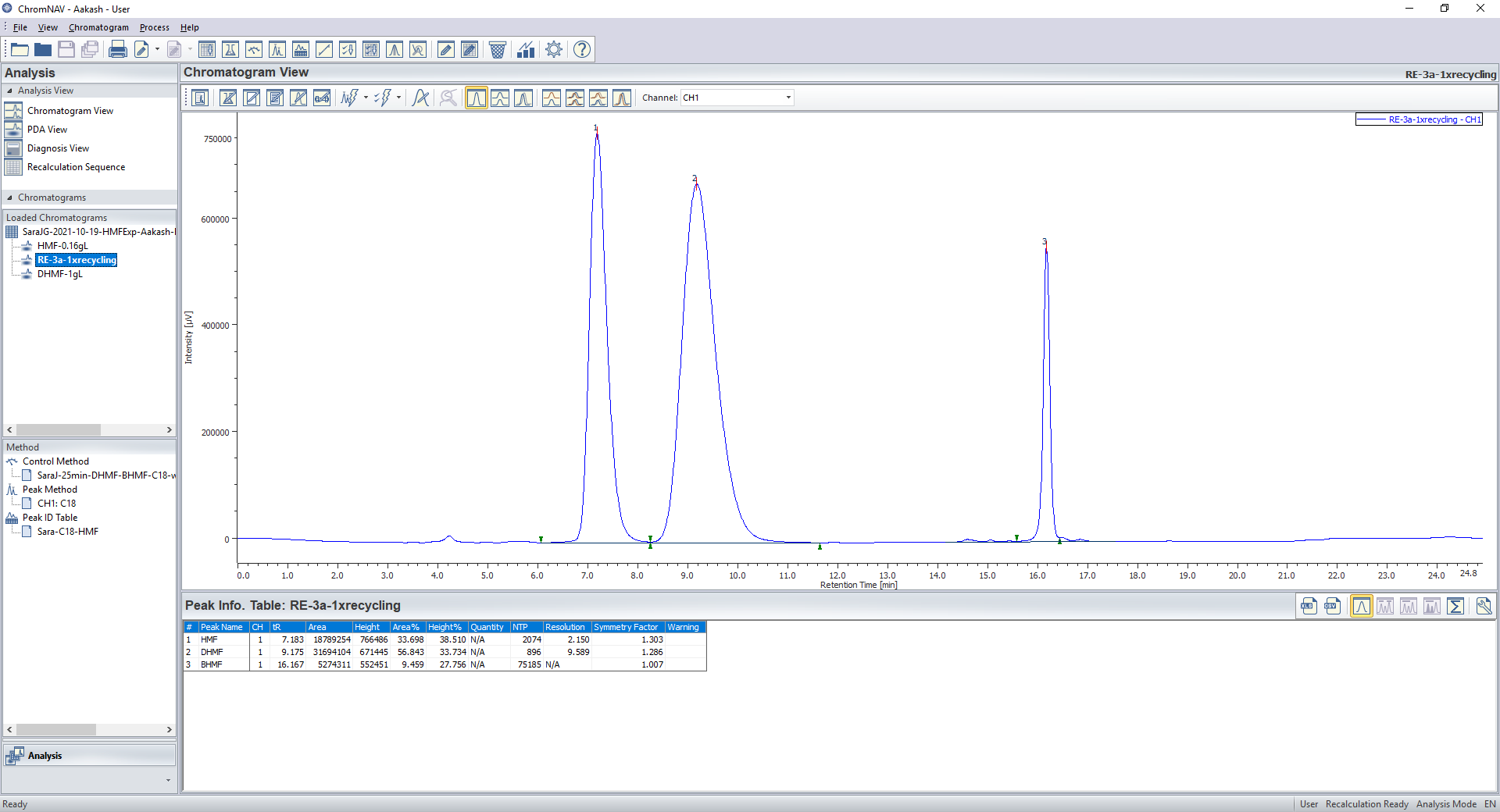


**Figure S6.** Representative chromatogram for quantitative analysis of 5-HMF, DHMF and BHMF by HPLC using reversed phase column (C_18_) and 20–80% methanol/water as mobile phase at 30°C, and UV detection at 280 nm. The retention times were 7.2 min, 9.4 min and 16.2 min for 5-HMF, DHMF anf BHMF, respectively.


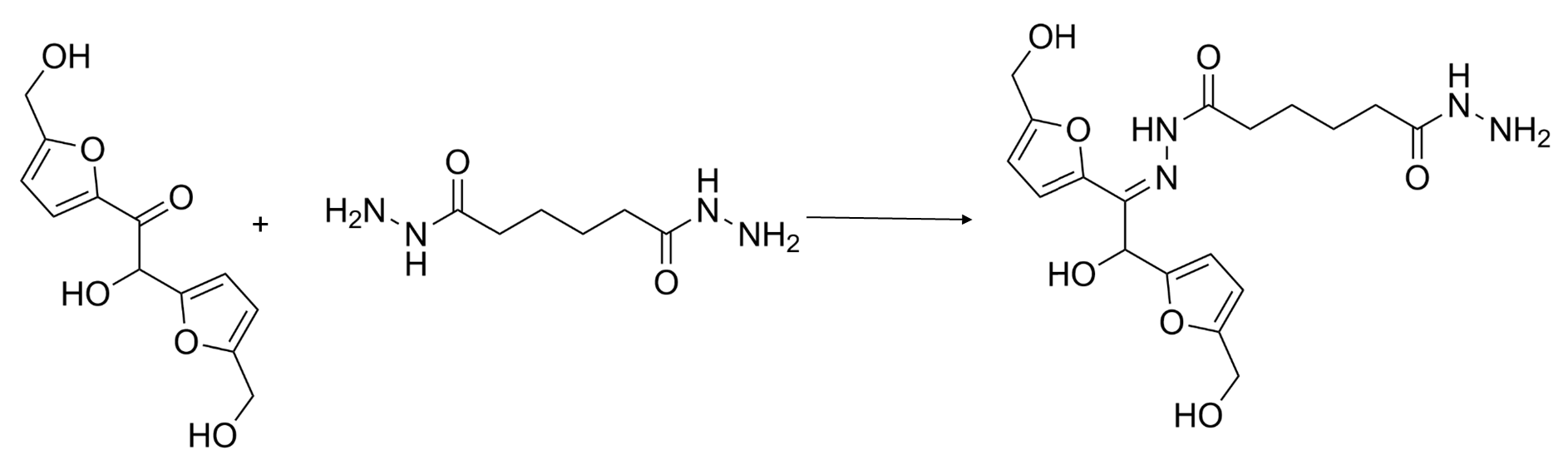


**a**


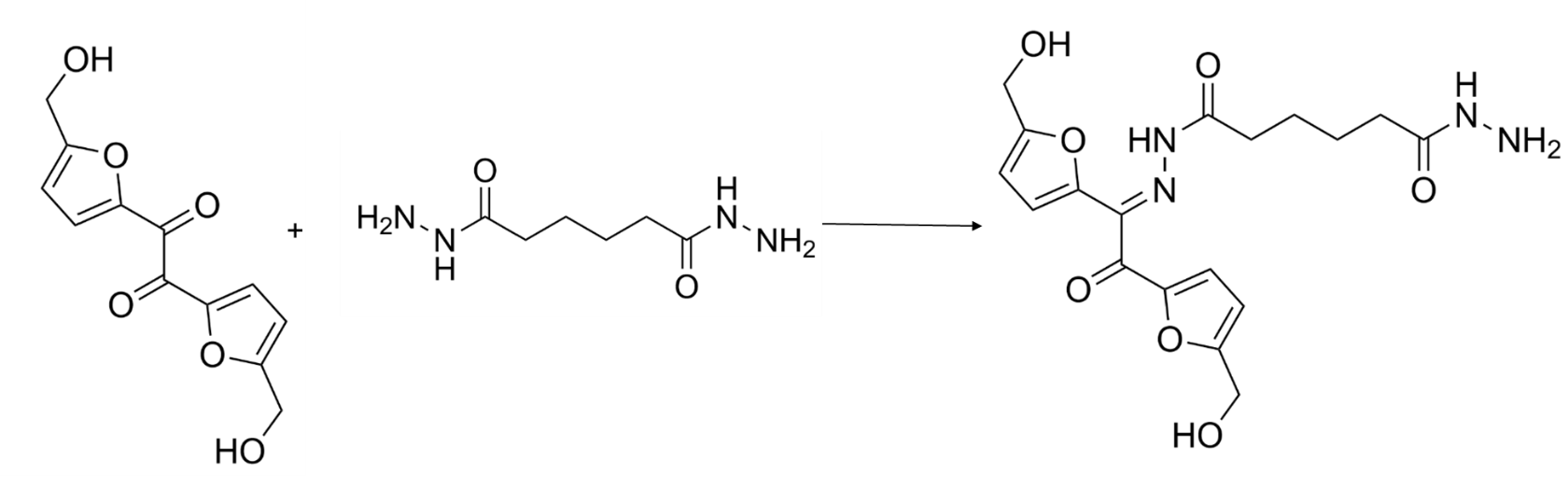


**b**

**Scheme S1.** Hydrazone formation via reaction with adipic dihyrazide. (a) DHMF-based hydrazone, and (b) BHMF-based hydrazone.
